# Appendicitis in Children: Evaluation of the Pediatric Appendicitis Score in Younger and Older Children

**DOI:** 10.1155/2014/438076

**Published:** 2014-12-10

**Authors:** Martin Salö, Gustav Friman, Pernilla Stenström, Bodil Ohlsson, Einar Arnbjörnsson

**Affiliations:** ^1^Department of Clinical Sciences, Lund University and Department of Pediatric Surgery, Skåne University Hospital, 221 85 Lund, Sweden; ^2^Department of Clinical Sciences, Lund University and Division of Internal Medicine, Skåne University Hospital, 205 02 Malmö, Sweden

## Abstract

*Background*. This study aimed to evaluate Pediatric Appendicitis Score (PAS), diagnostic delay, and factors responsible for possible late diagnosis in children <4 years compared with older children who were operated on for suspected appendicitis.* Method*. 122 children, between 1 and 14 years, operated on with appendectomy for suspected appendicitis, were retrospectively analyzed. The cohort was divided into two age groups: ≥4 years (*n* = 102) and <4 years (*n* = 20).* Results*. The mean PAS was lower among the younger compared with the older patients (5.3 and 6.6, resp.; *P* = 0.005), despite the fact that younger children had more severe appendicitis (75.0% and 33.3%, resp.; *P* = 0.001). PAS had low sensitivity in both groups, with a significantly lower sensitivity among the younger patients. Parent and doctor delay were confirmed in children <4 years of age with appendicitis. PAS did not aid in patients with doctor delay. Parameters in patient history, symptoms, and abdominal examination were more diffuse in younger children.* Conclusion*. PAS should be used with caution when examining children younger than 4 years of age. Diffuse symptoms in younger children with acute appendicitis lead to delay and to later diagnosis and more complicated appendicitis.

## 1. Introduction

Appendicitis is the most common abdominal disease requiring surgery in children [1]. The risk of developing appendicitis during a lifetime is reported to be 8.7% for boys and 6.7% for girls [2]. Despite its high incidence, there are still diagnostic difficulties. The overall negative appendectomy rate among all children is suggested to be 8.4%, but in children under 6 years of age, the rate has been reported to be as high as 56.7% [3]. The diagnosis of acute appendicitis is considered to be especially challenging in children due to difficulties in communication and examination [4]. There are several studies reporting difficulties in diagnosing appendicitis in younger children [3, 5–9]. The diagnostic difficulties result in increased risks of both negative appendectomies and a delayed diagnosis, both leading to increased morbidity, more complications, longer hospital stay, and higher costs [3, 5–9]. These risks are further increased in the younger children [3, 8, 9]. The doctor delay is a known cause contributing to late diagnosis in young children [5, 6]. Other studies, with patients under 3 years and 4 years of age, have found parent delay to contribute as well to the late diagnosis [7, 10]. Our clinical experience, confirmed by the literature, shows that the younger children with acute appendicitis deviate from the typical presentation and clinical findings observed in older children with acute appendicitis [6–9].

The use of a clinical score, based on patient history and examination, is one way to possibly improve the diagnostic procedure. There are several available scores, recently reviewed by Kulik et al. [11]. In this systematic review, the Alvarado score and the Pediatric Appendicitis Score (PAS) were considered the most reliable. PAS is the only score specifically developed for children, composed by Samuel [12] in 2002 when analyzing children between 4 and 15 years of age. PAS has been validated and recommended by some authors [13, 14], but only one of these studies has included children less than 4 years of age [14]. Further, no study has compared PAS between older and younger children.

We hypothesized that PAS could be helpful in diagnosing young children with appendicitis and that we would find both parent delay and doctor delay contributing to the often late diagnosis in this age group. The aims of this study were to (i) evaluate PAS in children <4 years of age in comparison with children ≥4 years old, operated on for suspected appendicitis, with respect to PAS sensitivity, specificity, positive predictive value (PPV), and negative predictive value (NPV); (ii) investigate if there was a delay in diagnosing appendicitis in children <4 years compared with older children; and (iii) identify factors responsible for the possible late diagnosis in younger children.

## 2. Material and Method

### 2.1. Settings and Children

The prospectively collected database of all children admitted to a tertiary center of pediatric surgery was used. The center serves an area with 1.8 million inhabitants and performs surgery on all children under 3 years of age. As the health care is free, any drop-out due to socioeconomic factors is unlikely. Surgeons have a permanent position at the center and perform surgeries during their regular working hours; surgeons are also paid for overtime on-call services. The preoperative evaluation and work-up were performed only by the surgeon who later operated on the child.

### 2.2. Study Design

This study is an institution-based, retrospective study. All children <15 years of age who underwent appendectomy between January 2010 and March 2014 were searched for using ICD-10 procedure codes (JEA00, JEA01, and JEA10). The endpoint of the study was the completion of the appendectomy and the following 30 days. Charts including notes from the operation, laboratory tests, radiology, and histopathological analysis were retrospectively studied. The diagnosis of appendicitis was based on operative findings and, in most cases, combined with the histopathological analysis.

Medical records were examined and the following characteristics were recorded: age, sex, time from onset of symptoms to seeking care (parent delay), how often the child was evaluated by a doctor and sent home without suspicion of appendicitis and without a rescheduled follow-up (doctor delay), which diagnosis was presumed in patients with doctor delay, presenting symptoms, notes from the abdominal examination, presence of leukocytosis and/or neutrophilia, type of radiology used, surgeon's description of the severity of the appendicitis, results from the histopathological analysis, days of in-patient care, and complications. With the information on patient history, abdominal examination, and laboratory tests, PAS was calculated for each patient. PAS consists of eight parameters: (1) migration of pain, (2) anorexia, (3) nausea/vomiting, (4) right lower quadrant (RLQ) tenderness, (5) cough/percussion/hopping tenderness in the RLQ, (6) elevated temperature, (7) leukocytosis, and (8) polymorphonuclear neutrophilia [12]. Each parameter is assigned 1 point except the physical signs (4 and 5) which are assigned 2 points, giving a maximum score of 10. A score ≥6 is said to indicate a high risk of appendicitis [12]. The patients were divided into two groups according to their age: ≥4 years of age and <4 years of age.

### 2.3. Statistical Considerations

The statistical analysis was performed by a statistician. Each child <4 years of age was compared with five children between 4 and 15 years of age. Prior data indicated that the probability of exposure among controls is 0.4. If the true probability of exposure among cases was 0.2, we would need to study 20 case patients and 100 control patients to be able to reject the null hypothesis that exposure rates for cases and controls are equal to a probability (power) of 0.8 [15]. The Type I error probability associated with the test of the null hypothesis was 0.05. SPSS Statistics was used for statistical calculations. Fisher's two-tailed exact test was used for dichotomous variables and the Mann-Whitney *U*-test for ranked results. A *P* value < 0.05 was considered statistically significant.

### 2.4. Ethical Considerations

The study was performed according to the Helsinki Declaration and approved by the Regional Ethical Review Board (registration number 2010/49). The data were anonymized prior to calculations and were presented in a way that made it impossible to identify any single patient.

## 3. Results

### 3.1. Patients

**Figure 1:**
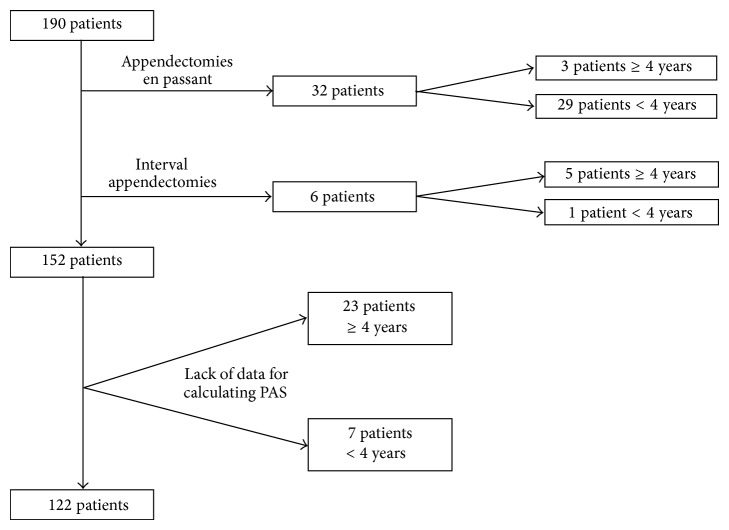
Flow chart of patient recruitment.

A total of 190 patients who underwent surgery were identified. Thirty-eight patients were excluded, due to the appendectomy being performed en passant or as an interval appendectomy, leaving a total of 152 patients to analyze. Of these, 30 patients were further excluded due to lack of data for calculating PAS. Seven of the excluded patients were <4 years of age, of whom six had appendicitis and one had a negative appendectomy. Twenty-three of the patients excluded were >4 years of age, 20 of which had appendicitis and three had a negative appendectomy (Figure 1). Thus, a total of 122 patients, 74 boys and 48 girls, were finally included in the study. There were 20 patients <4 years of age (mean 2.6 years) and 102 patients between the ages of 4 and 14 years (mean 10.5 years) (Table 1). The excluded patients did not differ in the severity of the appendicitis when compared with the included patients.

### 3.2. PAS

Mean PAS was significantly lower among the younger patients than among the older patients (Table 2). The sensitivity of PAS with a cutoff at six points was low in both groups but significantly lower in the younger group. With a cutoff at five points, no significant difference in sensitivity was observed. The specificity, PPV, and NPV varied with different cutoffs and between the two age groups. Generally, the specificity and PPV were high, and the NPV was low. The specificity among the younger patients with a cutoff at six points was 100% (Table 2). The PAS in patients with doctor delay showed lower values compared with patients without doctor delay. All patients under <4 years and with a doctor delay had a score <6 (Table 3).

### 3.3. Parent Delay and Doctor Delay

Parent delay and doctor delay were more common among children <4 years of age. On average, younger patients were brought for medical care 35 h later than older patients. Among younger patients, 25% were seen by a doctor at the pediatric emergency room (ER) and sent home without suspicion of appendicitis and without a scheduled readmission compared with 5.9% for older patients. Regarding doctor delay, unspecific abdominal pain was a more common diagnosis among older children than among younger children. The patients sent home had no specific treatment plan or scheduled follow-up, except for one patient with suspected pyelonephritis (Table 3).

### 3.4. Symptoms and Laboratory Data

There were no significant differences between the two age groups regarding the following symptoms and laboratory data: vomiting/nausea, anorexia, hopping/percussion/coughing, tenderness in the RLQ, urinary tract infection (UTI), leukocytosis, and neutrophilia (Table 4).

A significant difference between age groups was found for the following symptoms: fever ≥38°C (more common in younger children) and RLQ pain and history of typical pain migration (more common in older patients). All older patients sought medical care with abdominal pain as the main complaint and were triaged as acute abdomen compared with 85.0% of younger patients. After exclusion of those with appendiceal abscesses, the remaining younger patients had a higher frequency of diarrhea than the older patients (Table 4).

### 3.5. Radiology

Ultrasound (US) and/or computer tomography (CT) of the abdomen were used to the same extent in both groups. In the older group, 60.7% of patients were examined with US and 4.9% with CT. The corresponding figures for the young group were 75.0% and 5.0%, respectively. However, preoperative investigation with both methods was significantly more commonly used in younger children (20.0%) compared with older children (4.9%) (*P* = 0.039).

### 3.6. Grade of Inflammation and Negative Appendectomy

There was no significant difference between the two age groups regarding amount of gangrenous and perforated appendicitis and number of appendiceal abscesses (*P* = 0.154, 0.071, and 0.123, resp.). However, phlegmonous appendicitis was significantly more common in older children than in younger children, 59.8% compared to 10.0% (*P* = 0.000). When combining the more severe types of appendicitis (gangrenous, perforated, and appendiceal abscess), younger patients showed a significantly more severe inflammation, 75.0% compared to 33.3% (*P* = 0.001). The rate of negative appendectomies was higher among the younger children (15.0%) compared to the older children (6.9%), but not significantly different (*P* = 0.211).

### 3.7. Hospital Stay and Complications

No differences in postoperative complications such as abscess, wound infection, and intestinal obstruction were found. Younger patients had more days of in-patient care compared with older patients (Table 5).

## 4. Discussion

The PAS was significantly lower among the younger compared with the older patients, despite the fact that younger children had more severe appendicitis. Furthermore, PAS had low sensitivity in both groups with a significantly lower sensitivity among the younger patients. Both parent delay and doctor delay were found in younger children with appendicitis. Parameters in patient history, symptoms, and abdominal examination were more diffuse in younger children.

We hypothesized that PAS would be helpful in diagnosing acute appendicitis in younger children. However, the mean value of PAS at the time of the first doctor examination was significantly lower for younger children than for older children. The original study describing PAS did not include younger children [12]. To our knowledge, only one study evaluating PAS has included younger children. This study included patients between 1 and 17 years of age who sought medical care with a chief complaint of abdominal pain lasting less than 7 days [14]. In the same study, PAS was considered to be useful, with a PAS ≤ 2 ruling out appendicitis and a PAS ≥ 7 predicting appendicitis with high validity. It was difficult to make this comparison in our study because our cohort consisted of children who underwent appendectomy and not children seeking medical care for abdominal pain. Furthermore, the sensitivity of PAS was low in both groups, although significantly lower in the younger group. PAS sensitivity was lower when compared with other studies that analyzed cohorts of children with abdominal pain [13, 16, 17]. With a cutoff at ≥6, we found low specificity (14%) among older children and a much higher specificity (100%) among younger children. Because our cohort consisted of patients who underwent appendectomy due to suspected appendicitis, it is hard to draw any conclusions regarding the specificity of PAS from this study.

Considering that younger children had more severe appendicitis, a higher PAS value in this group would be expected. Because this was not the case, one could speculate about the reasons for such a finding. Regarding patient history, the younger patients “lose points” for not being able to describe pain migration. A history of typical pain migration was observed in 50% of the older patients but was absent among younger patients. This may be explained by the difficulty, among younger children, to localize and describe the pain. Regarding physical examination, tenderness in the RLQ was significantly less among younger patients, a finding that was also described by others [6, 7, 18]. One explanation may be that many of the children had perforated appendicitis at consultation, a condition that presents with more diffuse pain [6]. The difficulty for the younger children to describe pain migration, as well as the absence of RLQ tenderness, is thus a limitation in using PAS in young children because pain migration and RLQ tenderness are included in the score. On the other hand, fever was more common among younger children, largely due to a higher rate of severe appendicitis. This probably increased the mean PAS in the younger group, which raises the question of whether PAS would have been even lower if the groups were matched based on the severity of the appendicitis. There were no significant differences between younger and older children in terms of presence of leukocytosis and neutrophilia at consultation. However, this may be hard to evaluate considering that younger children had more severe appendicitis. To our knowledge, there are no previous studies reporting such a difference.

Moreover, two other parameters contributed to the diffuse clinical picture in the younger children: 85% of these children sought medical care with abdominal pain as the main complaint and were triaged as acute abdomen when compared with older children. Younger children with appendicitis who are not triaged as acute abdomen have been described by others as well [6]. Furthermore, even when excluding those with appendiceal abscesses, younger patients still had a higher frequency of diarrhea than older patients. This result is in concordance with other studies, and it may confuse the clinical picture and mislead the surgeon [19].

The fact that parent delay and doctor delay contribute to the late diagnosis of appendicitis in younger children was confirmed in our present study [5–7, 10]. Younger children associated with a doctor delay were presumed to have another specific diagnosis and not unspecific abdominal pain. This finding stresses the diffuse clinical picture that young children with appendicitis may present. PAS was exceptionally low in patients with doctor delay, especially regarding the younger children. Hence, PAS did not aid in the diagnosis. Furthermore, younger children had a significantly higher frequency of complicated appendicitis than older children at consultation. Our results are in agreement with previous studies that showed more severe appendicitis in younger children [5–7, 9]. We anticipate that parent delay and doctor delay largely explain the more severe appendicitis in younger children. Both delays can be explained by the younger children having more diffuse symptoms and difficulty in communication and during examination [4]. Not all appendicitis specimens were sent to histopathological analysis, which may have led to a misjudged grade of inflammation. However, one can assume that such bias should have occurred equally in the two groups.

## 5. Conclusion

The PAS scoring system turned out to be a weak tool in diagnosing appendicitis in children, especially in younger children. Furthermore, PAS did not aid in patients with doctor delay. Parent delay and doctor delay were confirmed as contributing factors in the delayed diagnosis of appendicitis in younger children, which may explain the higher rate of complicated appendicitis in this group. More studies, including prospective studies, of children with suspected appendicitis are needed, especially with a focus on younger children.

## Figures and Tables

**Table 1:** Patient demographics.

	≥4 years	<4 years
Patients (*N*)	102	20
Sex (male/female)	63/39	11/9
Age (mean (range), years)	10.5 (4–14)	2.6 (1–3)

Number of patients, sex, and age in the two age groups.

**Table 2:** Comparison of PAS in older and younger patients.

PAS	≥4 years *N* = 102	<4 years *N* = 20	*P* value
PAS (mean (range))	6.60 (2–10)	5.25 (2–9)	0.005^*^

PAS ≥ 5 (%)	Sensitivity: 87.3 Specificity: 14.2 PPV: 93.2 NPV: 7.6	Sensitivity: 70.5 Specificity: 66.7 PPV: 92.3 NPV: 28.5	0.085^**^

PAS ≥ 6 (%)	Sensitivity: 70.5 Specificity: 14.2 PPV: 91.7 NPV: 3.4	Sensitivity: 41.2 Specificity: 100.0 PPV: 100.0 NPV: 23.1	0.018^**^

PAS: Pediatric Appendicitis Score; PPV: positive predictive value; NPV: negative predictive value; ^*^Mann-Whitney *U*-test, two-tailed; ^**^Fisher's exact test, two-tailed.

**Table 3:** Parent and doctor delays.

	≥4 years	<4 years	*P* value
Time from onset of symptoms to seeking care (mean (range), hours)	36.4 (2–144)	70.8 (12–168)	0.005^*^
Prior doctor consultation^a^ without scheduled readmission (*n* (%))	6/102 (5.9)	5/20 (25.0)	0.017^**^
Presumed diagnosis in patients with doctor delay (*n*)	Unspecific abdominal pain (4) and constipation (2)	Gastroenteritis (2), pyelonephritis, constipation, and unspecified virus infection	
PAS in patients with doctor delay^a^ (mean (range))	4.8 (2–6)	3.8 (3–5)	0.317^*^

^a^Doctor at the pediatric emergency room (ER) sent the child home without any suspicion of appendicitis, and hence, no planned reevaluation of the child the next day; *n*: numbers; ^*^Mann-Whitney *U*-test, two-tailed; ^**^Fisher's two-tailed exact test.

**Table 4:** Symptoms and clinical findings in older and younger patients.

Finding	≥4 years *N* = 102	<4 years *N* = 20	*P* value^*^
Nausea/vomiting	72 (70.5)	13 (65.0)	0.605
Anorexia	89 (87.2)	16 (80.0)	0.478
Fever > 38°C	37 (36.3)	16 (80.0)	0.000
Migration of pain	49 (48.0)	0 (0)	0.000
Leukocytosis	65 (63.7)	12 (60.0)	0.802
Neutrophilia	81 (79.4)	15 (75.0)	0.766
RLQ tenderness	90 (88.2)	13 (65.0)	0.016
Hopping/percussion/ coughing and tenderness in RLQ	53 (51.9)	10 (50.0)	NA
Triaged as acute abdomen	102 (100)	17 (85.0)	0.004
Diarrhea	5 (4.9)	4 (20.0)	0.039
UTI symptoms	1 (0.9)	2 (10.0)	0.070

Values are given as the absolute number and percentage of patients. RLQ: right lower quadrant; UTI: urinary tract infection; NA: not applicable; ^*^Fisher's two-tailed, exact test.

**Table 5:** Hospital stay and complications.

	≥4 years *N* = 102	<4 years *N* = 20	*P* value
Days of in-patient care (mean (range), days)	3.1 (1–35)	7.5 (1–20)	0.000^*^
Complications (*n* (%))	13 (12.7)	4 (20.0)	0.478^**^

*N*: numbers; ^*^Mann-Whitney *U*-test, two-tailed; ^**^Fisher's exact test, two-tailed.

## Abbreviations

CT: Computed tomography
MRI: Magnetic resonance imaging
NPV: Negative predictive value
PAS: Pediatric Appendicitis Score
PPV: Positive predictive value
RLQ: Right lower quadrant
US: Ultrasound
UTI: Urinary tract infection.
